# Differences between brainstem gliomas in juvenile and adult rats

**DOI:** 10.3892/ol.2013.1319

**Published:** 2013-04-25

**Authors:** YU WANG, YONGJI TIAN, HONG WAN, DEZHI LI, WENHAO WU, LUXIN YIN, JIAN JIANG, WEIQING WAN, LIWEI ZHANG

**Affiliations:** 1 Department of Neurosurgery, Beijing Tian Tan Hospital, Capital Medical University, Beijing 100050, P.R. China; 2 Beijing Neurosurgical Institute, Beijing 100050, P.R. China

**Keywords:** brainstem, gliomas, rat, magnetic resonance imaging, survival

## Abstract

Clinical studies have shown that gliomas of the brainstem behave differently in children and adults. The aim of the present study was to compare and analyze the differences between these gliomas in juvenile and adult rats with regard to tumor growth, survival, pathology and magnetic resonance imaging (MRI). A total of 25 juvenile and 25 adult Wistar rats were divided into groups A (15 juvenile rats), B (10 juvenile rats), C (15 adult rats) and D (10 adult rats). The rats of groups A and C (experimental) were injected with glioma cells, while groups B and D (control) were injected with a physiological saline solution. Rat neurological signs, survival time, tumor size, hematoxylin and eosin (HE) staining and immunohistochemical staining for MMP-2, MMP-9 and β-catenin were compared. The survival time of group A was 19.47±2.232 days, whereas that of group C was 21.47±2.232 days (P<0.05). The tumor sizes were 4.55 and 4.62 mm (P>0.05) in groups A and C, respectively. HE and immunohistochemical staining revealed no differences between the groups. The results suggest that the growth patterns and invasiveness of brainstem gliomas may vary in children compared with adults due to the varied biological behaviors of the tumor cells.

## Introduction

Gliomas of the brainstem account for ∼10–20% of all central nervous system tumors (1). The present study retrospectively analyzed clinical data from the past 20 years and identified that brainstem gliomas (BSG) may be divided into four types, diffuse, intrinsic focal, exophytic focal and cervicomedullary, according to the tumor site. They may also be separated into diffuse infiltrative BSG, known for their relentless growth and poor outcome, and focal BSG, which are associated with a favorable prognosis. BSG occur most often in children aged 7–9 years and in the fourth or fifth decade of adult life (2,3). All BSG were previously regarded as malignant, since their location rendered them inoperable. However, as modern technologies in neuroimaging and microsurgery have developed, it has become possible to remove certain types of BSG by surgery. However, surgical intervention has only been beneficial in focal BSG (4). There are currently no effective therapeutic treatments for diffuse BSG (5–7). The development of a satisfactory experimental model for these gliomas is critical for understanding their biological behavior (8,9).

Numerous clinical studies have indicated that adult BSG vary from those of children (3,10–12). The majority of BSG in children are diffuse tumors, accounting for up to 80% of brainstem tumors in children. The tumors cause diffuse infiltration and swelling of the brainstem, with a clinical presentation that includes involvement of the sixth nerve, ataxia, cerebellar signs and long-tract signs in the form of a short prodrome of symptoms (<6 months), which indicate a worse prognosis. Surgery is not beneficial for these patients. Radiation has been shown to provide temporary stabilization or a transient improvement of clinical symptoms. The median survival time of children with diffuse BSG is <1 year following diagnosis. In adults, focal gliomas represent the majority of tumors, and these displace the long tract of the brainstem. The tumors tend to displace rather than infiltrate neural cells and have well-demarcated borders. Surgery may be performed to remove the tumor in this type of glioma. The median survival time is >4 years. Overall, BSG in adults are less aggressive and have a better prognosis than those in children (1).

Genetic abnormalities associated with BSG in children are different from those in adults, which may contribute to the differences in the tumor growth patterns. The differing anatomical characteristics may also contribute to the varied growth patterns. Whether tumor cells in children are more invasive than those of adults or if stronger nerve fibers in the adult brainstem are able to block the invasion of tumors has not been determined. The present study aimed to establish brainstem glioma models using juvenile and adult rats. The study also investigated the difference between BSG in juvenile and adult rats with regard to tumor growth, survival, pathology and magnetic resonance imaging (MRI).

## Materials and methods

### Animals

Female juvenile Wistar rats (3 weeks old; body weight, 40–50 g) and adult female Wistar rats (8 weeks old; body weight, 200–220 g) were purchased from the Institute of Laboratory Animals, Chinese Academy of Medical Sciences (Beijing, China). A C6 glioma cell line, originally cloned in N-nitrosomethylurea and in an F-12 medium containing 2.5% fetal bovine serum (FBS) and 15% horse serum (HoS), was purchased from the Institute of Basic Medical Sciences, Chinese Academy of Medical Sciences (Beijing, China). The main equipment used in the present study included a 3.0 Tesla MR machine and 5-inch surface coil (GE Healthcare, Stamford, CT, USA; and Sigma, St. Louis, MO, USA), an inverted phase contrast microscope (Nikon, Gotenba, Japan), a carbon dioxide (CO_2_) incubator (Heraeus, Hong Kong), a microtome (Leitz, Stuttgart, Germany), rat stereotaxic apparatus (Xibei Optical Instrument Factory, Shanxi, China), a microinjection pump (New Era Pump System, Inc., Farmingdale, NY, USA) and 10-*μ*l microsyringes (Feige, Shanghai, China). This study was carried out in strict accordance with recommendations of the Guide for the Care and Use of Laboratory Animals of the National Institutes of Health. The animal use protocol was reviewed and approved by the Institutional Animal Care and Use Committee (IACUC) of Beijing Neurosurgical Institute (permit number, 20060828001).

### Animal model

Wistar rats (25 adults and 25 juveniles) were divided into groups A (experimental group, 15 juvenile rats), B (control group, 10 juvenile rats), C (experimental group, 15 adult rats) and D (control group, 10 adult rats). The rats were injected with 1×10^5^ C6 glioma cells (groups A and C) or physiological saline solution (groups B and D). The treatment solutions were stereotactically injected into the pons of each rat. The C6 cells were cultured in an F-12 medium with 2.5% FBS and 15% HoS at 37°C in a humidified incubator containing 5% CO_2_ (13). Cell passaging was performed prior to injection and the culture fluid was replaced 24 h later. In the logarithmic phase with 80% cell confluence, the cells were digested with 2.5% trypsin. The action of trypsin was terminated by the addition of the medium when the majority of cells were round, the cell processes had retracted and the cell gaps were widening. The collected cell suspension was centrifuged at 500 × g for 10 min. Viable cells were identified by Trypan blue exclusion and counted with a hemocytometer. The cells were diluted to a concentration of 1.0×10^5^ cells/10 *μ*l.

### Surgical technique

The animals were anesthetized with 10% chloral hydrate administered intraperitoneally. The head of each rat was fixed on a stereotactic frame. The parieto-occipital skin was shaved and prepared in sterile conditions. A midline incision of ∼1 cm in length was made. The lambdoid suture was identified and a 1-mm burr was drilled 1.5 mm posterior to this and 1.5 mm to the left of the sagittal suture for the rats in groups A and B. A 1-mm burr was drilled 2.0 mm posterior to the lambdoid suture and 2.0 mm to the left of the sagittal suture in the rats of groups C and D. A 10-*μ*l microsyringe containing 1.0×10^5^ cells was fixed onto the microinjection pump. The needle was inserted into the burr hole to a depth of 8.5 mm from the bone surface in the adult rats and 7.5 mm from the bone surface in the juvenile rats. The suspended cells were injected at a speed of 1 *μ*l/min. Subsequent to the injection, the needle remained in place for 10 min to avoid a backflow of cells. The needle was then slowly withdrawn from the burr hole, which was closed with bone wax, then the skin was sutured (14,15).

### Observation of behavior and survival

All rats were weighed every day. Neurological deficits were observed and evaluated, including evaluation of the corneal reflect using a cotton swab on the eyes, as well as observation of movement to determine if the rats had weakness or hemiparesis of the legs. The survival time of each rat was recorded.

### MR scan

At two weeks post-implantation, the animals were anesthetized with 10% chloral hydrate administered intraperitoneally. The animals were then scanned in the axial, sagittal and coronal planes with a slice thickness of 1.5 mm. A T1-weighted image (T1WI) was scanned following an intravenous injection of gadolinium diethylenetriamine pentaacetic acid (Gd-DTPA; 0.4 ml/kg).

### Hematoxylin and eosin (HE) staining

One day after the MR scan, all animals were sacrificed using an intraperitoneal injection of an excessive dose of chloral hydrate and then perfused with 0.9% saline followed by 4% paraformaldehyde. Subsequent to the removal of the brainstem, fixation, paraffin embedding and slicing (5 *μ*m in thickness) were performed and the slides were stained with HE.

### Immunohistochemical analysis

The slides were deparaffinized, rehydrated and boiled in 10 mM citrate buffer (pH 6.0) for 10 min in an oven to expose the antigen. Non-specific binding sites were blocked by incubating the slices with 10% normal goat serum for 30 min at room temperature. The slides were incubated with primary antibodies (MMP-2, MMP-9 and β-catenin; 1:100 dilution; Millipore, Billerica, MA, USA) overnight at 4°C. The sections were then washed and incubated with a secondary antibody at room temperature for 30 min. Visualization was performed using the streptavidin-peroxidase method combined with 3,3′-diaminobenzidine. The integrated optical density (IOD) was calculated using medical image analysis software (ImagePro Plus, Media Cybernetics Inc., Bethesda, MD, USA).

### Statistical analysis

The statistical analysis was performed using SPSS 13.0 (SPSS, Inc., Chicago, IL, USA). The results are presented as the mean ± SD. The survival, tumor size and IOD of groups A and C were compared, and Kaplan-Meier survival curves and weight change curves were generated for the two groups. P<0.05 was considered to indicate a statistically significant difference.

## Results

### Behavioral observation

The rats in groups A and C presented with secretions from the left eye at 1 week post-implantation. The left corneal reflex was blunt compared with the right reflex, which indicated an extraocular muscle problem. Hemiparesis was also observed. The two groups of rats exhibited weakness, apastia and a gradual reduction in movement as the tumor grew. The rats then died. None of the rats in groups B or D exhibited abnormal behavior.

### Survival rate

**Figure 1.**
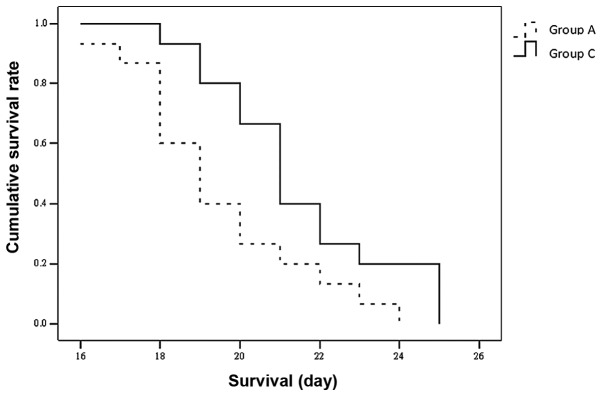
Survival time. Survival times of 16–24 days (mean, 19.47±2.232 days) and 18–25 days (mean, 21.47±2.232 days) were observed in groups A and C, respectively. The difference was statistically significant (P<0.05).

A total of 2, 3, 3 and 2 rats in groups A, B, C and D, respectively, died on the day of the implantation or 1 day post-operatively, due to epidural or subdural hemorrhage induced by the drilling process. The survival time for the remaining rats was recorded as 16–24 days (mean, 19.47±2.232 days) in group A and 18–25 days (mean, 21.47±2.232 days) in group C. The difference in the survival time of groups A and C was statistically significant (P<0.05; Fig. 1). All the rats in group A and C were alive at the time when all the rats in groups B and D had died.

### Weight observation

**Figure 2.**
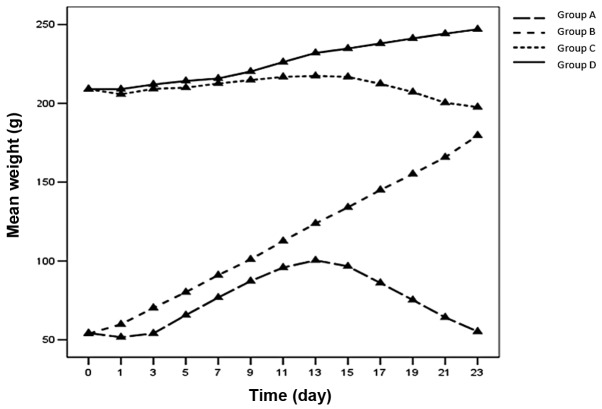
Weight gain. The rats in groups A and C exhibited a ‘decrease-increase-decrease’ mode of weight gain.

The weight of the rats in groups B and D continued to increase, although the increase was not as rapid as predicted by the age-matched control data on Wistar rats supplied by the Institute of Laboratory Animals. The rats in groups A and C exhibited a ‘decrease-increase-decrease’ mode of weight gain (Fig. 2).

### MRI scans

**Figure 3.**
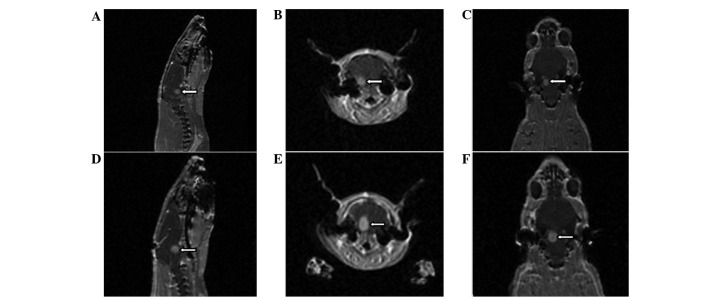
Tumors in (A–C) juvenile and (D–F) adult rats. The signals of the tumors were low in the T1WIs and high in the T2WIs. Following the injection of Gd-DTPA, the signal in the T1WIs was clearly enhanced, revealing necrosis inside the tumors. The tumors exhibited marked mass effects, resulting in ventricular dilation where the tumor was obstructing the pathway of the CSF. Arrows indicate the tumor. T1WI, T1-weighted image; Gd-DTPA, gadolinium diethylenetriamine pentaacetic acid; CSF, cerebrospinal fluid.

MRI scans were performed at 2 weeks post-implantation and tumor growth was confirmed. The tumors were round and confined within the pons, with a clear boundary. The tumor formation rate was 84.6% (11/13) in group A and 83.3% (10/12) in group C. The signals of the tumors were low in the T1WIs and high in the T2WIs. Following the injection of Gd-DTPA, the signal in the T1WIs was markedly enhanced and revealed necrosis inside the tumor. The tumors exhibited marked mass effects, which resulted in ventricular dilation since the tumors obstructed the pathway of the cerebrospinal fluid (Fig. 3). The tumor volume was calculated using the formula, V = 4/3 × πr^3^. The mean maximal diameter of the tumor was 4.55 mm in group A and 4.62 mm in group C (P>0.05).

### HE staining

**Figure 4.**
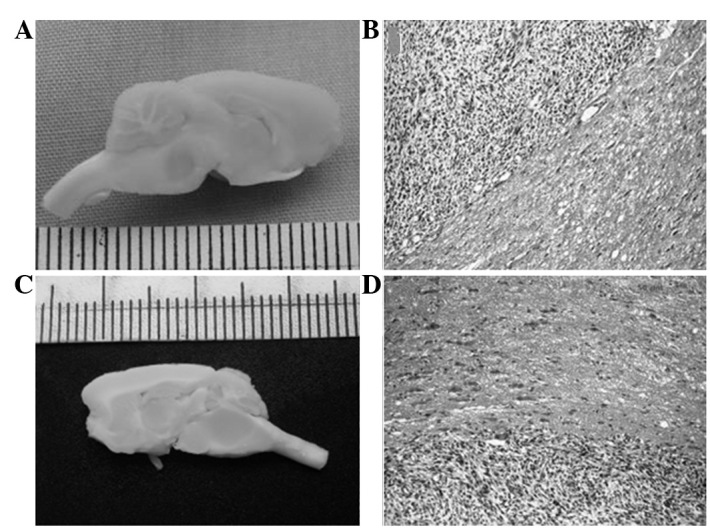
(A and C) Gross specimens of the juvenile and adult rats. The tumors were mass-like and there were significant differences with the surrounding normal tissue. (B and D) Invasive growth (HE; magnification, ×100). (A and B) represent group C and (C and D) represent group A. HE, hematoxylin and eosin.

The tumors were mass-like with relatively clear boundaries upon gross examination. Using HE staining, the boundary was evident without a membrane envelope, and intratumoral necrosis was also clearly observed. Tumor growth was characterized as invasive. The new vessels were abundant and palisade-like necrosis was evident. The tumor cells were active, exhibiting several shapes (including diamond, triangle and oval shapes) and showing an increased nucleus-cytoplasm ratio. HE staining revealed no significant differences between groups (Fig. 4).

### Immunohistochemical staining

**Figure 5.**
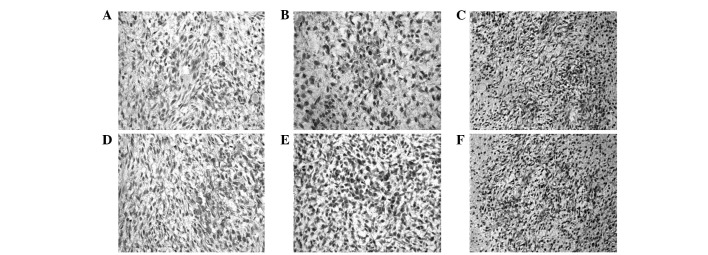
(A) MMP-2, (B) MMP-9 and (C) β-catenin protein expression in juvenile rats; (D) MMP2, (E) MMP-9 and (F) β-catenin protein expression in adult rats.

The MMP-2, MMP-9 and β-catenin staining in juvenile and adult rats was positive in the cytoplasm and nucleus (Fig. 5). The number of positive cells and expression levels (IOD value) were not significantly different between groups (Table I).

## Discussion

In clinical studies, gliomas of the brainstem exhibit marked differences in children and adults with regard to incidence rate, form of onset, progression speed and prognosis (16,17). The prognosis of diffuse tumors in children is usually poor, whereas the prognosis for the focal type typically observed in adults is better (18). Studies have attempted to investigate whether this difference is caused by the biological behavior of the tumor cells or by intensive nerve fibers and nuclei in the adult brainstem, which block invasion of the tumor. However, the answer this conundrum remains unknown. In the present study, the proliferation and invasion of BSG were compared in juvenile and adult rats using morphology and imaging anayses. The main factors that determine the different features of BSG in the two age groups were also explored.

With the growth of the tumors, the nerve nuclei and cranial nerves (V, VI, VII and VIII) in, or derived from the brainstem were compressed. Rats in groups A and C exhibited a blunt corneal reflex in the left eye, which suggested an impaired function of the extraocular muscles. Hemiparesis resulting from motor fiber damage was also observed. The rats exhibited gradually increasing weakness, apastia and eventually died.

Several rats bled when the needle pierced the dura mater. They died the same day or the day after implantation, possibly due to epidural or subdural hemorrhage. Rats in groups A and C received the same quantity of cells; however, the survival of the adult rats was longer than that of juvenile rats (P<0.05). This difference in survival may be due to the larger size of the adult rats. In juvenile rats, the space within the skull is smaller than that of adult rats. Therefore, the compensatory space in the skulls of juvenile rats is likely to be limited compared with that in adult rats, as the tumor volumes increase at a similar speed and size in all rats. This causes the tumor to compress the brainstem more severely in juvenile rats. Additionally, in the present study, hydrocephalus appeared earlier in the juvenile rats, which resulted in shorter survival times. This is consistent with the results from a study by Liu *et al* (19).

Rats in groups A and C exhibited a ‘decrease-increase-decrease’ pattern of weight gain, which is in contrast with the data reported by Liu *et al* (19). Subsequent to implantation, the stress response following surgery and anesthesia caused a short-term reduction in eating and drinking, which resulted in weight loss. As rats recovered from the stress response, they began to eat and drink normally again, thus increasing their weight. However, the rate of increase was lower than that of the control group. With the growth of the tumors, the rats exhibited apastia and their weight began to decrease. The weight of the juvenile rats at the time of death was heavier than at the beginning of the study, whereas the adult rats weighed less at the time of death compared with their baseline weight.

The majority of studies on BSG in animal models focus on pathology and survival (20–22). In the present study, MRI was used to evaluate tumor growth for the first time. This approach permitted observation *in vivo*, therefore, the continuous observation of growth was possible. The tumors of rats in groups A and C were ball-like and had a low signal in the T1WIs and a high signal in the T2WIs and the T1WIs enhanced by Gd-DTPA. These imaging results are similar to those reported for glioblastomas in humans (23).

HE staining showed that the tumor cells grew actively and were densely arrayed with evident mitosis. The growth was invasive with palisade-like necrosis. These characteristics are similar to those exhibited in human glioblastoma (24). However, no differences were observed between the juvenile and adult rats. The tumors in all rats were focal rather than diffuse. Therefore, the present study was unable to obtain conclusive results on the morphological differences between the two age groups. MMP-2, MMP-9 and β-catenin were also used to test for differences between adult and juvenile rats for the first time. Positive staining for all three antibodies was observed in the cytoplasm and nuclei in all sections; however, the difference between the two age groups was not statistically significant. In summary, MRI and HE and immunohistochemical staining were performed in the present study, however, no statistically significant morphological or image-based differences were identified between the BSG in the juvenile and adult rats. This may be due to the use of the same tumor cell type in the two groups of rats.

Clinical studies on BSG have demonstrated differences between children and adults. From the results obtained in the present study, we conclude that the differences are likely to be caused by the heterogeneity of the tumor cells themselves, rather than by the nerve fibers and nuclei in the adult brainstem blocking tumor invasion. Different types of cells should be used in future experiments. Tumor cells from the BSG of children and adults should be acquired, cultured and purified as two different cell lines to establish an immunodeficient animal model.

## Figures and Tables

**Table I.** IOD values of MMP-2, MMP-9 and β-catenin in juvenile and adult rats.

Group	MMP-2	MMP-9	β-catenin
A	46.99±8.23	71.18±12.53	100.48±9.11
C	48.13±9.02	66.28±14.42	103.69±7.50

Data are expressed as mean ± SD. IOD was tested using medical image analysis software. The values are similar between groups A and C, however, the difference was not statistically significant (P>0.05). IOD, integrated optical density.
